# Duzhong Butiansu Prescription Improves Heat Stress-Induced Spermatogenic Dysfunction by Regulating Sperm Formation and Heat Stress Pathway

**DOI:** 10.1155/2020/6723204

**Published:** 2020-02-27

**Authors:** Ying Hou, Peipei Yuan, Yang Fu, Qi Zhang, Yaxin Wei, Liyuan Gao, Li Liu, Xiaolan Wang, Xiaoke Zheng, Weisheng Feng

**Affiliations:** ^1^Henan University of Chinese Medicine, Zhengzhou 450046, China; ^2^Guizhou HanFang Pharmaceutical Co., Ltd., Guizhou 550081, China; ^3^Co-construction Collaborative Innovation Center for Chinese Medicine and Respiratory Diseases by Henan & Education Ministry of China, Zhengzhou, China

## Abstract

**Background:**

Duzhong Butiansu (DZBTS) prescription contains many traditional Chinese medicines and has been shown to have a curative effect on male fertility. However, the efficacy and mechanism of DZBTS in the treatment of male infertility induced by heat stress have not been reported. The aim of the present study is to elucidate the effect and mechanism of DZBTS on spermatogenic function of a heat stress model in rats.

**Methods:**

Male Wistar rats (280–320 g) were given different doses of DZBTS (0.4853 g/kg/d or 0.9707 g/kg/d), Shengjing capsule (0.56 g/kg/d), or double distilled water for 15 days. A 43°C hot water bath for 30 minutes was used to stimulate the testis of rats. Sperm count, sperm motility, the organ index of kidney and gonadal organs, serum sex hormone levels, and serum oxidising reaction index were measured. Haematoxylin and eosin (HE) staining was used to observe the morphology of the testis and kidney. The expression of Hsp70 in testes was observed by immunofluorescence. The changes in heat stress, reproductive-related protein, and mRNA were measured by western blot assay and RT-qPCR.

**Results:**

Heat stress downregulated the levels of sex hormone (*P* < 0.05 or *P* < 0.05 or *P* < 0.05 or *P* < 0.05 or *P* < 0.05 or *P* < 0.05 or *P* < 0.05 or

**Conclusions:**

Our results for the first time have found that DZBTS can improve spermatogenesis disorder in a heat stress model in rats, which may be mainly by regulating AR, sperm regulatory protein CREB1, and the HSF/Hsp70 signaling pathway to decrease oxidative stress.

## 1. Introduction

Heat stress response (HSR) refers to the stress reaction of the body in a high-temperature environment. It has been found that the mechanism of temperature regulation in the male scrotum in high-temperature environments will be damaged, resulting in compromised sperm quality and viability [1]. Some studies have shown that testicular hyperthermia can induce spermatogenic cell apoptosis, affect sperm development, and decrease sperm concentration, resulting in spermatogenic disorder [2]. However, in daily life, more and more men are in high temperatures for short or long periods of time due to their occupations [3] (e.g., sedentary desk workers [4], welders [5], drivers [6], and construction workers [7]). The use of complementary and alternative medicines has been a concern. Currently, Western medicine (e.g., gonadotropin replacement therapy, dopamine receptor agonists, and antioxidant supplements) has been used to treat spermatogenesis disorders [8]. In recent years, traditional Chinese medicines have been gradually developed to treat spermatogenic disorders (e.g., Qiangjing decoction, Bushen Shengjing prescription, Jinkui Shenqi pill, and Shengjing capsule). However, there are few reports to clarify the mechanism of Chinese medicine used to treat the spermatogenesis disorder induced by heat stress.

Duzhong Butiansu (DZBTS) prescription, which has been approved by the China Food and Drug Administration (license number: YBZ13402009), contains *Eucommia ulmoides* Oliv., *Cuscuta australis* R. Br., *Cistanche deserticola* Y. C. Ma, *Epimedium brevicomu* Maxim., and other traditional Chinese medicines for tonifying the kidneys and reinforcing yang [9], as well as *Angelica sinensis* (Oliv.) Diels., *Rehmannia glutinosa* Libosch., *Dioscorea opposita* Thunb., and other traditional Chinese medicines for tonifying the blood and qi [9]. The effect of the preparation of warming the kidney and nourishing the heart is recorded in the *Pharmaceutical Standards of the Ministry of Health of the People's Republic of China Traditional Chinese medicine prescription (Volume 12)*. The traditional Chinese medicine prescription, which works by tonifying the kidneys and reinforcing yang and tonifying the blood and qi, has a therapeutic effect on sperm dysfunction [10] and contains medicines such as Bushen Huoxue decoction [11], Shugan Yiyang capsules [12], and Yiqi Zhujing granules [13, 14]. The therapeutic effect on spermatozoa dysfunction of most of the traditional Chinese medicine contained in the prescription has also begun to be discovered, such as *Cuscuta australis* R. Br. [15], *Morinda officinalis* How [16], and *Epimedium brevicomu* Maxim [17]. After nearly 10 years of clinical observation and patient feedback, it was found that this prescription treated male oligospermia, asthenospermia, nonliquefaction of the seminiferous tubules, and other symptoms. However, the mechanism of its treatment for spermatogenesis disorder caused by heat stress has not been reported. The therapeutic effect and mechanism of DZBTS on male infertility induced by heat stress are discussed in this article. A model of spermatogenesis disorder in rats induced by heat stress was established. The sperm number and motility, sex hormone level, organ index, oxidative injury, and sex hormone receptor were observed. The effects of DZBTS on sperm formation, heat stress-related protein, and their RNA content were evaluated in this study to provide a theoretical basis for the clinical treatment of male infertility induced by heat stress with DZBTS.

## 2. Materials and Methods

### 2.1. Animals

Seven-week-old male (280–320 g) Wistar rats (SPF) were purchased from Beijing Vital River Laboratory Animal Technology Co., Ltd. The animal license number was SCXK (Beijing) 2016-0006. The rats had free access to rodent chow and drinking water in the SPF grade animal facilities with 23°C ± 2°C temperature and a 12-hour light/dark cycle. The experiment was approved by the Animal Ethics Committee of Henan University of Chinese Medicine.

### 2.2. Drugs

The DZBTS was provided by Guizhou Hanfang Pharmaceutical Co., Ltd. The prescription has been approved by the China Food and Drug Administration, license number: YBZ13402009.  Table 1 lists the 25 herbs in the prescription. *Nelumbo nucifera* Gaertn. and *Dioscorea opposita* Thunb. were ground to a fine powder. Water was added six times into *Citrus reticulata* Blanco and *Amomum villosum* Lour., and these were extracted for 3 hours to obtain the volatile oil. Water was added into the dregs, *Eucommia ulmoides* Oliv., and other herbs, 12 times, and they were boiled twice for 1.5 hours each time. The decoction was concentrated to a relative density of 1.5 to 1.18 and then cooled. Ethanol was added to the decoction in the amount of 1.5 times, followed by stirring evenly and setting overnight. Ethanol in the supernatant was recovered. The liquid was concentrated to a relative density of approximately 1.30. The fine powder of *Nelumbo nucifera* Gaertn. and *Dioscorea opposita* Thunb. was added. The mixture was mixed, dried, and crushed. The volatile oil was sprayed into the mixture and mixed. SJc was provided by Zunyi Liaoyuanhetang Pharmaceutical Co., Ltd. All drugs were dissolved with distilled water.

### 2.3. Animal Grouping and Administration

The male Wistar rats were divided into five groups according to their body weight: normal control group (NC, *n* = 11), model group (M, *n* = 11), positive control Shengjing capsule (0.56 g/kg/d) group (SJc, *n* = 11), DZBTS low-dose group (DZ-L: 0.4853 g/kg/d, *n* = 11), and high-dose group (DZ-H: 0.9707 g/kg/d, *n* = 11). The rats in each group were given 10 mL/kg of medicine soup by gavage once a day for 15 days. The normal control group and the model group were given the same dose of double-steamed water.

### 2.4. Model Preparation and Sampling

After administration, 10% sodium pentobarbital (Henan Colon Pharmaceutical, Henan) was injected intraperitoneally (40 mg/kg body weight). The rats were fixed, and the testes were placed in a water bath at 43°C for local heat stress (30 min) to establish the thermal stimulation model. After the model was established, the blood was taken immediately from the abdominal aorta. The kidneys, testes, and epididymis were weighed. One kidney and testis were immersed in formalin; the other epididymis was immediately evaluated for sperm quality. The kidney, testis, and epididymis were weighed and then frozen in liquid nitrogen and stored at −80°C.

### 2.5. Haematoxylin-Eosin Staining (HE) Staining

The kidney and testis were soaked in formalin for 2 days, and then paraffin sections were prepared and stained by HE staining. The HE solution is alkaline, which causes the chromatin in the nucleus and the nucleic acid in cytoplasm to be purple-blue. Eosin is an acid dye, which mainly causes the components in the cytoplasm and the extracellular matrix to be red. The changes in the testis and kidney tissue structure were observed. At the same time, the effect of DZBTS on the pathological changes of the testes and kidneys in heat stress rats was evaluated.

### 2.6. Determination of Sperm Content and Motility

The epididymis was taken from one side and added to 1 mL of saline. After shredding, the epididymis was incubated at 37°C for 8 minutes and then filtered by a 70-*μ*m filter membrane (BD Company, USA) with 4 mL of saline. Next, 20 *μ*L of the filtrate was dropped onto the slide, and the sperm motility was evaluated on the sperm quality analyser. Another 100 *μ*L of filtrate was added to 5 mL 5% NaHCO_3_ for fixation, and 20 *μ*L of fixed solution was used to count the sperm.

### 2.7. Determining the Level of Serum Sex Hormone and Oxidative Stress in Rats

The blood samples of rats in each group were centrifuged for 10 minutes at 3000 rpm and 4°C, and the serum was obtained. The enzyme-linked immunosorbent assay (ELISA, Elabscience Biotechnology Co., Ltd., Wuhan, China) double-antibody sandwich method was used to detect the binding of the rat follicle-stimulating hormone (FSH) and rat luteinizing hormone (LH). The sample or standard was added and bound to the corresponding antibody on the carrier. Then, the biotinylated antibody was added and bound specifically to the antigen, which was tested on the carrier. Horseradish peroxidase-labelled avidin and biotin were specifically combined to form an immune complex, which developed a colour and detected the optical density (OD) value. The levels of estradiol (*E*_2_) and testosterone (*T*) in the serum of rats were determined by the competitive method (Elabscience Biotechnology Co., Ltd., Wuhan, China). Samples or standard samples, horseradish peroxidase-labelled antibodies, and anti-antibodies were added to form complexes with the secondary antibodies on the carrier to colour and detect OD values. The activity of superoxide dismutase (SOD) was determined by the WST-1 (water soluble tetrazolium salt-1) method. WST-1 was reduced to purple dye, and this reaction was inhibited by SOD. The content of malondialdehyde (MDA) was determined by TBA (thiobarbituric acid). MDA in lipid peroxide degradation products can be condensed with TBA to form red products. GSH-Px (glutathione peroxidase) promoted the oxidation of GSH, and GSH reacted with dithiodidinitro benzoic acid to form yellow. Finally, the enzyme activity of SOD and GSH-Px and the content of MDA were calculated by colourimetric analysis (SOD, MDA, and GSH-Px from Nanjing Jian Cheng Bioengineering Institute, Nanjing, China).

### 2.8. Effect of DZBTS on Heat Stress and Reproduction-Related Protein Detected by Western Blot

PMSF (phenylmethanesulfonyl fluoride) and RIPA (radio immunoprecipitation assay) lysis buffer (1 : 1000, Beyotime Biotechnology, Shanghai, China) were added to the testes of each group of rats. These homogenates were centrifuged for 5 minutes at 4°C, 12000 g. The supernatant was extracted, and the protein was quantified by BCA Protein Assay Kit (Beijing ComWin Biotech Co., Ltd., Beijing, China). The supernatant was diluted with the 5× loading buffer according to the volume ratio of 4 : 1, and the sample concentration of each group was 60 *μ*g/10 *μ*L after 1× loading buffer supplementation. The gel plate was placed in the system of electrophoresis buffer solution, and the sample was added to the gel for electrophoresis. At the end of electrophoresis, according to the PageRuler™ Prestained Protein Ladder (Thermo Fisher Scientific, Shanghai, China) instruction, the gel of the target protein was retained. When the PVDF membrane (Millipore Immobilon P, USA) was activated with anhydrous ethanol, the membrane was infiltrated in the transposed solution. Imprinted with the protein by the semidry method, the PVDF membrane was rinsed by PBS (phosphate buffer saline) and then sealed in 5% skimmed milk powder for 1 hour. The androgen receptor rabbit antibody (1 : 1000, Abcam), CREB1 rabbit antibody (1 : 1000, Proteintech Group), HSF1 rabbit antibody (1 : 1000, Proteintech Group), Hsp70 mouse antibody (1 : 1000, Abcam), and *β*-actin mouse antibody (1 : 5000, Cell Signaling Technology) were incubated overnight at 4°C. The PVDF membrane was washed by PBST for 5 times and then put in the IRDye® 680 RD Goat anti-Rabbit (1 : 30000, LI-COR, USA) and IRDye® 800CW Goat anti-Mouse (1 : 30000, LI-COR, USA) for 1 hour. Next, the membrane was cleaned by PBST for 3 times. The ODYSSEY CLx double infrared laser imaging system was used to analyse the protein bands at 680 nm and 800 nm, and the protein expression was analysed according to the fluorescence intensity.

### 2.9. Real-Time Polymerase Chain Reaction (RT-PCR)

Lysate was added to the 100 mg testis of rats in each group. The RNA was extracted with a total RNA extraction kit (Solarbio, Beijing, China). The extracted samples were detected by a limited protein accounting analyser, and the RNA was reversed to cDNA by the Hiscript ΙΙ 1st Strand cDNA Synthesis Kit (Vazyme Biotech, Nanjing, China). Finally, the QuantiNova™ SYBR Green PCR Kit (QIAGEN, Germany) was used to detect the fluorescence quantitative PCR (qPCR) (Table 2).

### 2.10. Statistical Processing

The data were analysed by SPSS 18.0 software, and the data were expressed in the form of mean ± standard deviation (SD). Single-factor analysis of variance (ANOVA) was used to compare the differences between groups. The statistically significant differences between groups were determined by one-way ANOVA. *P* < 0.05 or *P* < 0.01 was considered to be statistically significant.

## 3. Results

### 3.1. Effect of DZBTS on the Visceral Index of Kidney and Gonadal Organs in Heat Stress Rats

**Figure 1:**
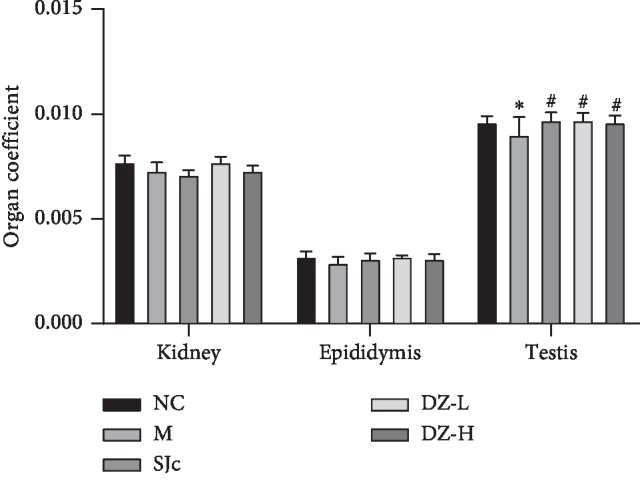
The organ coefficients of the kidney and sexual organs in rats. After weighing the kidney and sexual organs of each group, the ratio of organ to rat body weight was calculated. Values are expressed as means ± SD (*n* = 8; ^*∗∗*^*P* < 0.01, ^*∗*^*P* < 0.05 compared with the control; ^##^*P* < 0.01, ^#^*P* < 0.05 compared with the model).

Heat stress is a physical factor, and immersion time is short; thus, there is no significant change in the organ index, but the trend of testicular change has already appeared. After heat stress, the visceral coefficients of the kidney, epididymis, and testis of rats decreased and there was a tendency to improve the organ coefficient of testis after administration of DZBTS. The results suggest that DZBTS may have a tendency to improve the testis of heat stress rats (Figure 1).

### 3.2. Effect of DZBTS on Spermatogenic Function of Heat Stress Rats

**Figure 2:**
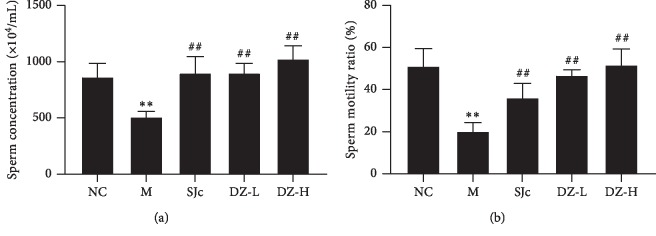
Effect of DZBTS on spermatogenic function of heat stress rats. (a) Sperm concentration. The diluted semen was dripped onto the slide, and more than three visual fields were selected under the microscope to observe the sperm count and take the average value for data analysis. (b) Sperm motility ratio. Semen drops are place onto the slide, and more than three visual fields are selected under the microscope. According to the Fifth Edition of Manual of Human Semen Testing and Processing Laboratory, the percentage of the sum of PR and NP in the total sperm count was observed. Values are expressed as means ± SD (*n* = 6; ^*∗∗*^*P* < 0.01, ^*∗*^*P* < 0.05 compared with the control; ^##^*P* < 0.01, ^#^*P* < 0.05 compared with the model).

The concentration and motility of spermatozoa decreased significantly after heat stress (*P* < 0.01), whereas DZBTS significantly increased the concentration and motility of sperm (*P* < 0.01). This suggested that DZBTS could significantly reduce the damage of spermatogenic function induced by heat stress (Figure 2).

### 3.3. Effect of DZBTS on Sex Hormone in Heat Stress Rats

Heat stress decreased the level of *T*, *E*_2_, FSH, and LH (*P* < 0.05 or *P* < 0.01), and DZBTS could increase the level of sex hormone (*P* < 0.05 or *P* < 0.01). The results indicated that DZBTS could upregulate the level of sex hormone in heat stress rats and improve its spermatogenic function (Table 3).

### 3.4. Effect of DZBTS on Oxidative Stress in Rats with Heat Stress

Heat stress could decrease the enzyme activity of SOD and GSH-Px and increase the content of MDA in the serum of model rats (*P* < 0.05 or *P* < 0.01). After administration of DZBTS, the enzyme activity of SOD and GSH-Px in serum was increased (*P* < 0.05 or *P* < 0.01) and the content of MDA in serum was decreased (*P* < 0.01). This indicated that DZBTS could reduce oxidative damage induced by heat stress in rats (Table 4).

### 3.5. Effect of DZBTS on Testicular and Renal Tissue Structure in Heat-Stressed Rats

**Figure 3:**
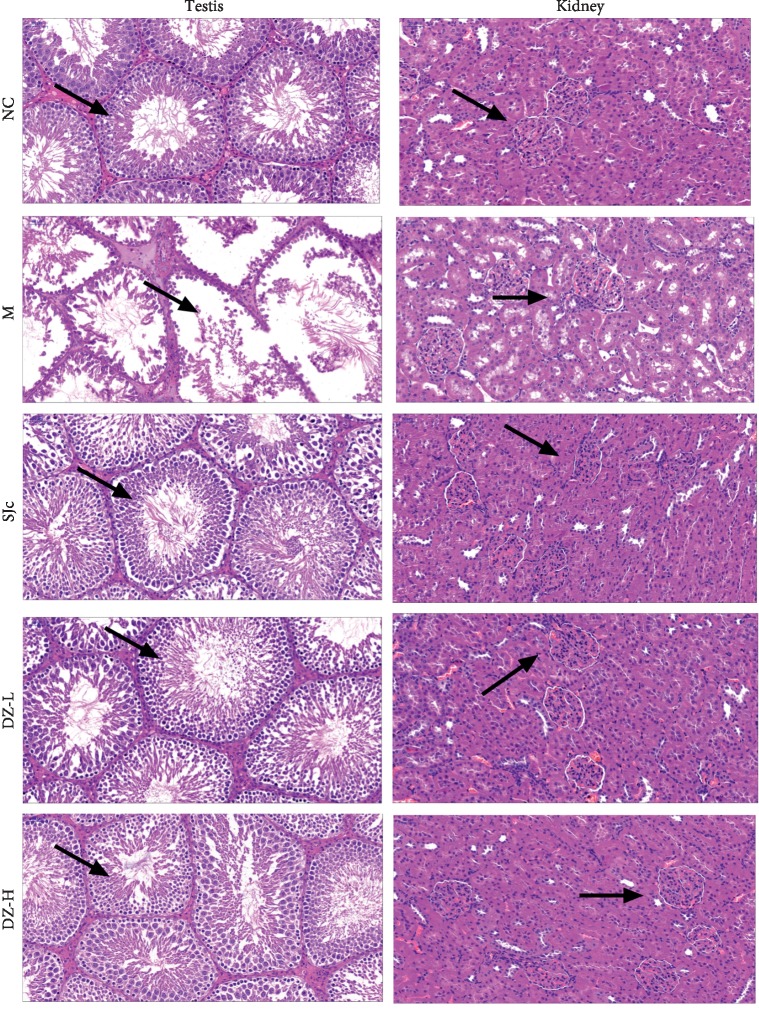
Effect of DZBTS on the tissue structure of testis and kidney in heat stress rats (HE, ×200, *n* = 3). The testis and kidney in NC, M, SJc, DZ-L, and DZ-H groups were stained by the HE method, and the pathological changes of organs were observed under a 200x microscope. The arrowheads in testicular sections refer to all levels of spermatogenic cells, and the arrows in renal sections refer to renal tubules.

As shown in Figure 3, the morphology of the testis and convoluted tubule in the normal control group was well developed, and the tubules were filled with spermatogenic cells. In the model group, heat stress induced the deformation of the lumen structure of the testis, induced the atrophy of the seminiferous tubules, disturbed the arrangement of spermatogenic cells, reduced the number of cells, and hindered the production of sperm, whereas, DZBTS could prevent the tubules of testicular seminiferous tubules from heat stress damage and fill the spermatogenic cells and spermatogenesis of the tubules. The results suggest that DZBTS could improve the spermatogenic function by improving the structure of the testis of heat stress rats (Figure 3).

As shown in Figure 3, the glomerular structure and the distribution of renal tubules in the normal control group were well formed. In the model group, there were no obvious changes in the glomeruli, slight atrophy of renal tubules, and slight hollowness. This result may be due to acute heat stress, and the effect on the structure of the kidney was not obvious. The glomeruli and renal tubules of the DZBTS group grew well. It was suggested that heat stress had a slight effect on the changes of the renal structure in rats and that DZBTS could improve the mild structural changes (Figure 3).

### 3.6. Effect of DZBTS on Heat Stress and Reproductive-Related Protein in Heat Stress Rats

**Figure 4:**
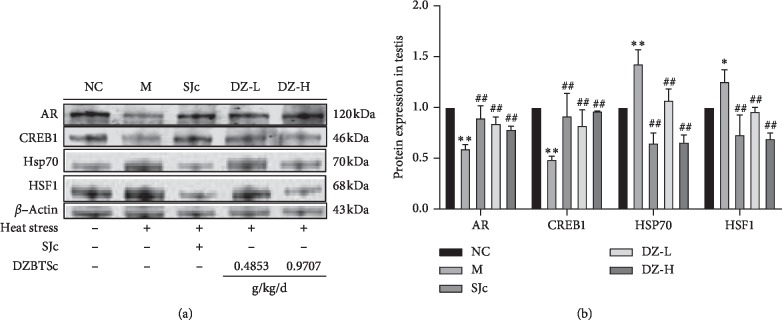
Effect of DZBTS on related proteins in the testis of heat stress rats. (a) Protein expression in the testis of heat stress rats. (b) Grey value of protein expression in the testis of heat stress rats. The PVDF membrane containing the protein band is imaged on the ODYSSEY CLx double infrared laser imaging system. Then, the protein bands were analysed by Image Studio Ver 5.2 software. Values are expressed as means ± SD (*n* = 3; ^*∗∗*^*P* < 0.01, ^*∗*^*P* < 0.05 compared with the control; ^##^*P* < 0.01, ^#^*P* < 0.05 compared with the model).

Heat stress downregulated the levels of the CREB1 protein (*P* < 0.01) which was involved in spermatogenesis. Meanwhile, the expression of the AR protein was decreased (*P* < 0.01). Then, the binding rate of *T* to its receptor (AR) became lower, and the production of sperm was reduced. Heat stress could upregulate the expression of the HSF1 protein (*P* < 0.05 or *P* < 0.01) and then affect the HSF1/Hsp70 protein pathway. DZBTS could reverse the decrease of CREB1 and AR expression (*P* < 0.05 or *P* < 0.01) and increase the receptor action rate. DZBTS could also inhibit the activation of the HSF1/Hsp70 pathway (*P* < 0.01) and reduce the oxidative damage of spermatozoa. These results suggest that DZBTS may reverse the damage of seminiferous function induced by heat stress by regulating the CREB1, AR, and the HSF1/Hsp70 signaling pathway (Figure 4).

### 3.7. Effect of DZBTS on Hsp70 Protein in Heat Stress Rats

**Figure 5:**
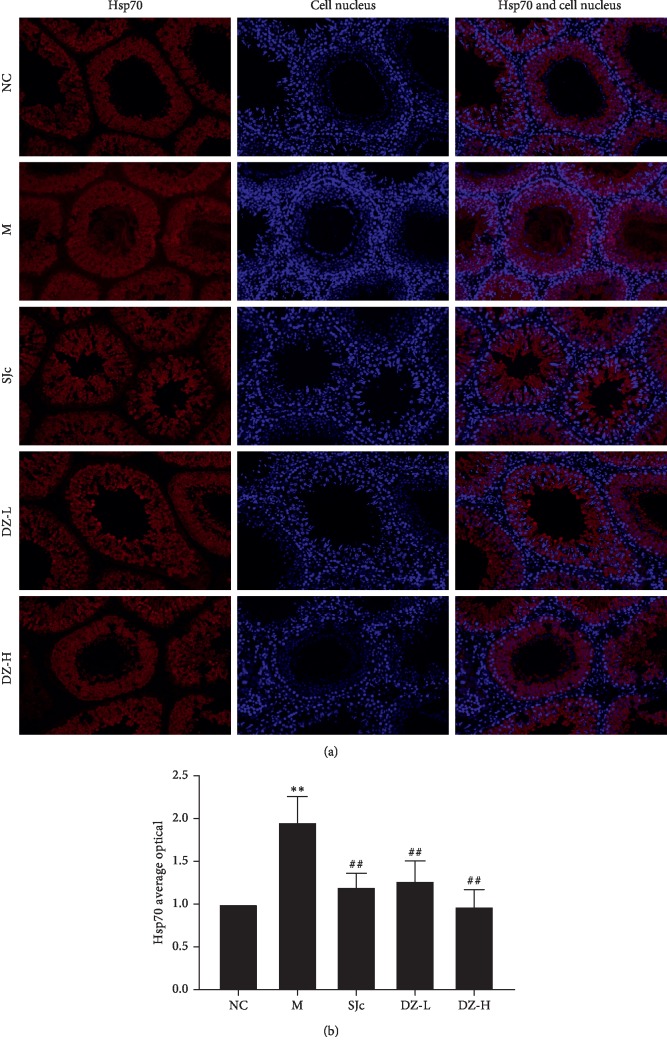
Effect of DZBTS on the expression of Hsp70 in the testis of heat stress rats (×200). (a) Hsp70 protein expression in the testis of heat stress rats. (b) Hsp70 average optical density. Each slice randomly selected more than three fields of view in 200 times the mirror to take pictures. Image-pro Plus 6.0 software was used to analyse each photo. Then, the cumulative optical density (IOD) and the pixel (area) of tissue were obtained. Average optical density value (AO value) is calculated. Values are expressed as means ± SD (*n* = 3; ^*∗∗*^*P* < 0.01, ^*∗*^*P* < 0.05 compared with the control; ^##^*P* < 0.01, ^#^*P* < 0.05 compared with the model).

The expression of Hsp70 protein in the testis of heat-stressed rats was detected by the immunofluorescence assay, and the results were quantitatively analysed. The results showed that heat stress could significantly increase the expression of Hsp70 (*P* < 0.01), and DZBTS could significantly reduce the expression of Hsp70 (*P* < 0.01). It was further verified that DZBTS may improve spermatogenic function of heat stress rats by regulating the expression of heat shock proteins (Figure 5).

### 3.8. Effect of DZBTS on mRNA in Heat Stress Rats

**Figure 6:**
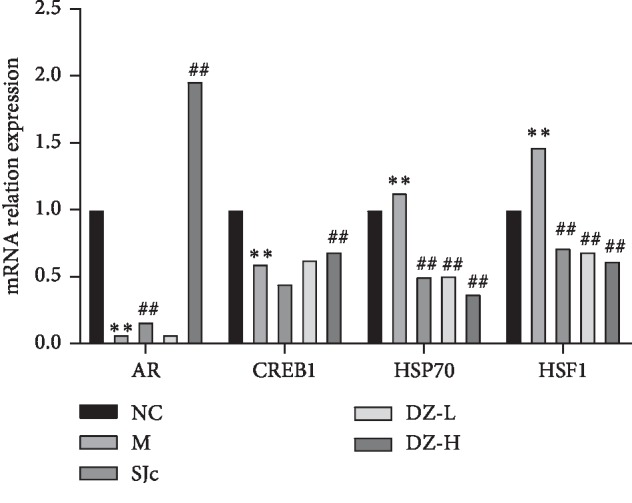
Effect of DZBTS on mRNA expression in the testis of heat stress rats. The cycle threshold (Ct) value of each sample was measured by the RT-qPCR, and the change of the Ct value in each group was analysed. Values are expressed as means ± SD (*n* = 3; ^*∗∗*^*P* < 0.01, ^*∗*^*P* < 0.05 compared with the control; ^##^*P* < 0.01, ^#^*P* < 0.05 compared with the model).

The mRNA level of AR, CREB1, HSF1, and Hsp70 in the testis of heat stress rats was measured by qPCR. The results were similar to the protein results. DZBTS could upregulate the mRNA level of AR and downregulate the mRNA level of HSF1 and Hsp70 (*P* < 0.01). There was also an upward trend of CREB1 mRNA level after administration. These results suggest that the drug can reverse the changes of spermatogenic function induced by heat stress by regulating sperm formation and androgen binding rate to its receptor and reducing the oxidative damage of spermatozoa (Figure 6).

## 4. Discussion

The normal growth and development of spermatozoa require suitable internal and external environments. When in low temperature or high temperature, the extreme temperature could lead to male fertility decline and even infertility [18]. The temperature of the testes is lower than the body temperature, and the normal survival temperature of human sperm is lower than the body temperature of 2°C–8°C [19]. If the temperature is higher than 1°C-2°C for a long period of time, it can cause hormonal disorders in the body. High temperature could increase the level of oxidative damage in vivo and cause changes in the microenvironment, hormone level, oxygen metabolism, and enzyme activity in the testis, resulting in weak sperm, oligozoospermia, and other spermatogenic disorders [20]. We found that heat stress significantly decreased the testis coefficient, sperm concentration, and motility in rats. Testicular morphology was changed by heat stress. These phenomena were significantly improved by DZBTS. Therefore, it was found that DZBTS could improve spermatogenic dysfunction induced by heat stress.

Sex hormones in the body regulate sperm production and increase sperm concentration. Studies have shown that the hypothalamus-pituitary-gonadal axis can affect spermatogenic function [21, 22]. The hypothalamus secretes gonadotropin-releasing hormone on the pituitary gland, and FSH and LH secreted from the anterior pituitary lobe can promote spermatogenesis [23]. FSH contributes to the division, proliferation, and differentiation of spermatogonia in the testes. LH stimulates testicular stromal cells to secrete *T*. Some studies have shown that *T* could be transformed into *E*_2_ to act on the estrogen receptor [24]. *E*_2_ was involved in the proliferation and differentiation of germ cells into mature spermatids, and it is important to testicular functions [25]. *T* regulates sperm production by actively transporting or diffusing into the vas deferens and by binding to the AR in Sertoli cells [26]. At the same time, *T* can promote the maturation of spermatozoa in the epididymis, promote the development of gonadal organs, and maintain its normal physiological function [27]. Furthermore, heat stress reduces intratesticular *T*, including androgens and estrogens [28]. Our study showed that heat stress reduced the levels of *T*, FSH, LH, and *E*_2_ in serum of rats, whereas all these conditions could be reversed by DZBTS, indicating that DZBTS could enhance the spermatogenic function.

It has been found that under heat stress, the testes exhibit many mechanisms, such as heat shock response and oxidative stress reaction [29]. Reactive oxygen species (ROS) produced by metabolism are important factors of sperm oxidative damage [30]. Under the action of high temperature, the body reacts and produces peroxides. When the ROS produced by the body exceeds the ability of the body to clear the peroxide, it can cause lipid peroxidation of the sperm membrane and damage the growth, development, quantity, and morphology of spermatozoa, which leads to infertility [31, 32]. Lipid peroxide, MDA, could indirectly reflect the degree of oxidative damage [33]. SOD could reduce the apoptosis of spermatogenic cells and play a role in sperm fertilisation potential and male infertility [34]. GSH-Px can restore the physiological state of unsaturated fatty acids on the cell membrane [35]. Thus, the oxidative damage of ROS to the cell membrane was alleviated. We found that heat stress significantly decreased the level of sex hormone and increased the level of oxidative injury in rats. The treatment of DZBTS could regulate the related indexes of oxidative stress and reduce the oxidative injury of the testes caused by heat stress.

**Figure 7:**
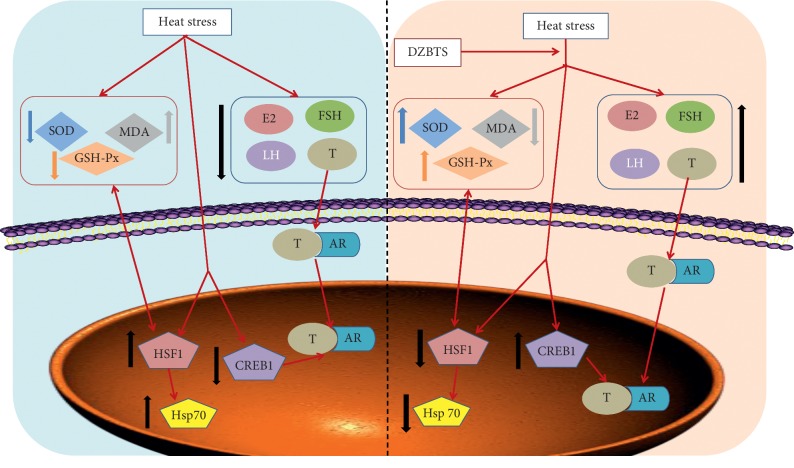
Signaling pathway of DZBTS in improving spermatogenic function.

Spermatogenesis was promoted by the combination of androgen with AR in the cytosol and closely related to the spermatogenesis-related proteins (CREB1) [36]. Androgen plays an important role in the initial completion and maintenance of sperm formation. Androgen deficiency or loss of functional AR can lead to spermatogenesis and infertility [37]. Studies have shown that heat stress inhibits the expression of AR in rats [36]. The expression of CREB1 [38] in spermatocyte mitosis and spermatogenesis is an important molecular regulatory factor for testicular development and spermatogenesis [39]. Moreover, CREB1 can stimulate the transcription of AR and promote the formation of spermatozoa [40]. Studies have shown that heat stress inhibits the expression of CREB1 mRNA and protein. The heat shock transcription factor (heat shock factor, HSF) [41] binds to the corresponding promoter in the heat stress response and initiates the transcription process of the gene, finally promoting the expression of the heat shock protein family (Hsps). The expression of Hsp that increased in the high-temperature environment [42] could urge the body to change into the stress mode and resist the sperm oxidative damage by temperature. Heat stress induced the increased expression of Hsps. Studies have shown that heat stress induced a significant increase in Hsp70 expression in mice [43]. Our findings show that heat stress significantly decreased the expression of sex hormone-related protein and upregulated the expression of heat stress-related protein. DZBTS may improve the spermatogenic function of heat stress rats by regulating the expression of sex hormone and heat stress-related protein, as shown in Figure 7.

## 5. Conclusion

Our results for the first time found that DZBTS can increase the binding rate of the androgen receptor by upregulating the sex hormone level and androgen receptor expression and promote the transcriptional activity of the androgen receptor complex in the nucleus by upregulating the transcription enhancer factor CREB1. In addition, DZBTS downregulated heat stress-related protein to reduce oxidative damage, improving the spermatogenic function of heat stress rats. These results indicate that DZBTS could be used for the diagnosis and treatment of male infertility induced by heat stress.

## Figures and Tables

**Table 1:** The composition of Duzhong Butiansu prescription.

Scientific name	Chinese name	Weight (g)	%
*Eucommia ulmoides* Oliv.	Duzhong	31.25	3.69
*Cuscuta australis* R. Br.	Tusizi	31.25	3.69
*Cistanche deserticola* Y. C. Ma	Roucongrong	31.25	3.69
*Polygala tenuifolia* Willd.	Yuanzhi	31.25	3.69
*Angelica sinensis* (Oliv.) Diels.	Danggui	31.25	3.69
*Nelumbo nucifera* Gaertn.	Lianzi	31.25	3.69
*Alisma orientale* (Sam.) Juzep.	Zexie	31.25	3.69
*Paeonia suffruticosa* Andr.	Mudanpi	31.25	3.69
*Paeonia suffruticosa* Andr.	Baishao	31.25	3.69
*Epimedium brevicomu* Maxim.	Yinyanghuo	28.125	3.32
*Astragalus membranaceus* (Fisch.) Bge.	Huangqi	62.5	7.38
*Rehmannia glutinosa* Libosch.	Shudihuang	62.5	7.38
*Dioscorea opposita* Thunb.	Shanyao	62.5	7.38
*Poria cocos* (Schw.) Wolf	Fuling	62.5	7.38
*Atractylodes macrocephala* Koidz.	Baizhu	62.5	7.38
*Citrus reticulata* Blanco	Chenpi	15.625	1.85
*Amomum villosum* Lour.	Sharen	15.625	1.85
*Ligustrum lucidum* Ait.	Nvzhenzi	14.06	1.66
*Rosa laevigata* Michx.	Jinyingzi	14.06	1.66
*Cornus officinalis* Sieb.et Zucc.	Shanzhuyu	3.125	0.37
*Morinda officinalis* How	Bajitian	3.125	0.37
*Platycladus orientalis* (L.) Franco	Baiziren	3.125	0.37
*Codonopsis pilosula* (Franch.) Nannf.	Dangshen	62.5	7.38
*Lycium barbarum* L.	Gouqizi	62.5	7.38
*Glycyrrhiza uralensis* Fisch.	Gancao	31.25	3.69

**Table 2:** List of primers and their sequences used in this study.

Gene	Forward primer	Reverse primer
AR	ATCAAGCTGGAGAACCCGTC	CTAGCCAAGTCCCCATAGCG
CREB1	CGGCCCAGCCATCAGTTATT	GCCTCCTTGAAAGGATTTCCC
HSF1	ATGCCATGGACTCCAACCTG	TCATGTCGGGCATGGTCAC
Hsp70	CCAGTGCGGCCTTAGTAGAG	CCTCAGACTCCGCCTTGTTT
GAPDH	ACAGCAACAGGGTGGTGGAC	TTTGAGGGTGCAGCGAACTT

**Table 3:** Effect of DZBTS on the level of sex hormone in heat stress rats $\left(\bar{x}\pm s,n=6\right)$.

Groups	*E*_2_ (pg/mL)	FSH (ng/mL)	LH (mIU/mL)	*T* (ng/mL)
NC	55.71 ± 2.94	12.16 ± 2.09	11.77 ± 1.63	0.69 ± 0.31
M	51.03 ± 2.27^*∗*^	8.98 ± 0.72^*∗*^	7.47 ± 1.84^*∗∗*^	0.16 ± 0.03^*∗∗*^
SJc	55.56 ± 3.04^#^	12.41 ± 0.52^##^	13.11 ± 2.71^##^	0.33 ± 0.15
DZ-L	63.02 ± 3.47^##^	11.75 ± 3.73^#^	11.72 ± 2.37^##^	0.45 ± 0.22^#^
DZ-H	63.86 ± 2.95^##^	12.28 ± 1.66^#^	16.81 ± 2.76^##^	0.38 ± 0.13

The OD values of each group were tested for analysis. Values are expressed as means ± SD (*n* = 6;^*∗∗*^*P* < 0.01, ^*∗*^*P* < 0.05 compared with the control;^##^*P* < 0.01, ^#^*P* < 0.05 compared with the model).

**Table 4:** Effect of DZBTS on the level of sex hormone in heat stress rats $\left(\bar{x}\pm s,n=5\right)$

Groups	SOD vitality (U/mL)	MDA (nmol/mL)	GSH-Px (IU)
NC	49.95 ± 13.16	6.89 ± 1.6	1440.68 ± 52.43
M	28.69 ± 8.42^*∗∗*^	11.22 ± 1.38^*∗∗*^	1280 ± 159.01^*∗*^
SJc	43.28 ± 4.73^#^	7.56 ± 1.08^##^	1416.17 ± 53.61^#^
DZ-L	51.06 ± 4.84^##^	7.78 ± 0.68^##^	1443.41 ± 45.93^#^
DZ-H	46.40 ± 4.31^##^	8.22 ± 2.31^##^	1447.49 ± 102.42^#^

The OD values of each group were tested for analysis. Values are expressed as means ± SD (*n* = 5;^*∗∗*^*P* < 0.01, ^*∗*^*P* < 0.05 compared with the control;^##^*P* < 0.01, ^#^*P* < 0.05 compared with the model).

## Data Availability

The data used to support the findings of this study are available from the corresponding author upon request.
